# Research on automatic emergency steering collision avoidance and stability control of intelligent driving vehicle

**DOI:** 10.3389/frobt.2023.1120658

**Published:** 2023-02-21

**Authors:** Zhaoyong Liu, Gaobo Wen, Wudong Liu, TanXiaoqiang Tan, Guangqiang Wu

**Affiliations:** ^1^ School of Automotive Studies, Tongji University, Shanghai, China; ^2^ Global Technology Co., Ltd, Nantong, China

**Keywords:** trajectory planning, stability control, LQR lateral control, emergency collision avoidance, direct yaw moment control

## Abstract

In view of the need for emergency steering to avoid collision when the vehicle is in a dangerous scene, and the stability control of the vehicle during collision avoidance. This paper proposes a planning and control framework. A path planner considering the kinematics and dynamics of the vehicle system is used to formulate the safe driving path under emergency conditions. LQR lateral control algorithm is designed to calculate the output steering wheel angle. On this basis, adaptive MPC control algorithm and four-wheel braking force distribution control algorithm are designed to achieve coordinated control of vehicle driving stability and collision avoidance safety. The simulation results show that the proposed algorithm can complete the steering collision avoidance task quickly and stably.

## Introduction

The Advanced Driver Assistance Systems (ADAS) are effective in reducing crashes. Most ADAS systems have one thing in common, that is, they all affect the longitudinal control of the vehicle to avoid the collision (Borrello et al., 2020; Rabhi et al., 2021; Zha et al., 2021; Hang et al., 2022; Wu et al., 2023).

Although, there are certain situations where a collision cannot be avoided by braking but only by steering operations. However, lots of studies show that in the case of an impending rear-end collision, many drivers tend to only brake rather than try to avoid obstacles by steering (Schieben et al., 2014). There are different reasons for this behavior. Firstly, it is an instinctive reaction to stop in order to reduce the impact of an impending collision. Secondly, steering is more complex than braking, so it requires the driver to have a higher awareness of the situation and a higher driving ability. Therefore, Automatic Emergency Steering System (AES) is of great significance.

For vehicle trajectory planning, Yang proposed a dynamic planning method for vehicle collaborative trajectory planning under the scenario of forced lane change, which aims to provide suggested lane change distance and reference trajectory for each autonomous vehicle in a coordinated manner (Yang et al., 2022). A dynamic programming way is established to determine the suggested distance of all vehicles and the non-convex quadratic constraint is applied to characterize the trajectory determination problem. Considering driver comfort and collision risk, Li et al. (2022) proposed a human-like motion planning strategy based on probabilistic prediction under dynamic environment. They realized path generation based on quintic polynomials, and optimized the target trajectory by using cost functions with four indexes including safety, consistency, smoothness and distance from local path to global path. Cheng et al. (2022) proposed a deep reinforcement learning method based on time difference to solve the longitudinal trajectory planning of autonomous vehicles at signal-controlled intersections. Xiao et al. (2021) [10] proposed the control barrier function method for critical safety control and developed a real-time control framework that combines the optimal trajectory generated with the computational efficiency method that provides safety assurance.

Vehicle collision risk assessment is the key to trigger AES. For vehicle risk assessment, Li et al., 2021 proposed a multi-scene collision avoidance decision algorithm for autonomous vehicles, and used the situation assessment module based on conditional random field to assess the risk level of the surrounding traffic participants. Based on the situation assessment module, collision avoidance strategies with driving style preferences (such as aggressive or conservative) are proposed to meet the needs of different drivers. Cui et al. (2021) proposed a layered framework of manned or autonomous vehicles for collision avoidance in emergency situations. They adopted finite-state machine (FSM) technology to determine appropriate strategies for collision avoidance, and established a collision risk model, taking into account vehicle risks around overlapping areas, road attachment risks and vehicle stability performance. Gilbert et al. (2021) proposed a decision-making system that selects the lightest collision when vehicles are confronted with inevitable collisions on the highway. They applied the multi-attribute decision-making method to judge the severity of the collision. For the autonomous lane change decision of trucks, Chen et al. (2020) proposed a lane change decision model based on support vector machine. Ren and Wu, 2020 proposed a fusion architecture of decision planning under dynamic Environment And Used Back Propagation Neural Network (BPNN) to predict the lane change of vehicles around the block.

The AES control layer will track the trajectory planned by the decision layer. For the vehicle trajectory tracking, Ge et al., 2022 rely on the precise model for the traditional MPC. When the autonomous vehicle encounters external interference and perturbation, the steady-state non-offset tracking cannot be realized, and the MPC solver is biased to solve the coupling control problem. Li et al. (2021) studied that under extreme driving conditions, the coupling between the longitudinal and transverse motion of the vehicle becomes significant due to the highly non-linear force of the tire, which affects the stability of the vehicle. They proposed a model prediction controller of electric vehicle driven by four-wheel independent motor, in which changes in the longitudinal velocity are regarded as interference in the vehicle dynamics model. Then, the additional torque generated by the model-based controller with the multi-objective design is considered for balance. Tork et al. (2021) proposed an independent model control based on neural network for path tracking control. The control scheme utilized the input of steering Angle and torque to realize cooperative control of transverse and longitudinal motion.

In summary, collision avoidance and stabilization are the two critical issues when an autonomous vehicle in an emergency situation, which usually occurs in a short time and requires large actuator inputs, as well as a highly non-linear response. Real-time vehicle decision-making, planning and control plays an important role in avoiding collisions while stabilizing autonomous vehicles in extreme scenarios. Liu et al. (2017) proposed a method to establish the stability criterion of vehicle yaw based on the phase plane method of sideslip-yaw rate, which solved the problem of judging the type of vehicle stability region under different driving conditions, and provided a theoretical basis for the intervention algorithm of stability control system. Zhang et al. (2017) considered the influence of tire slip and actuator torque saturation on driving and braking, and designed a dynamic controller to overcome integral saturation by using a conditional integrator to ensure accurate tracking of the required signals under the influence of tire force and actuator constraints. Vehicle state and parameter estimation is an important part of vehicle dynamic control. Liu et al. (2021) proposed a new estimation method of vehicle side-slip angle based on kinematic model, which integrated the information of Global Navigation Satellite System (GNSS) and inertial Measurement Unit (IMU). Xia et al. (2018) proposed a method to estimate the attitude and lateral velocity of an autonomous vehicle with the assistance of vehicle dynamics using a six-degree-of-freedom IMU. Liu et al. (2018) proposed a method based on kinematics model that integrates intelligent vehicle sensors to estimate sideslip angle, aiming at the problem that the non-linear characteristics and parameter uncertainties of vehicles make it difficult for the method based on dynamic model to estimate the sideslip angle of vehicles under harsh working conditions. Xiong et al. (2020) proposed a new automatic vehicle sideslip angle and attitude estimation method based on IMU for low sampling rate GNSS speed and position parallel adaptive Kalman filters.

The existing steering collision avoidance system often only considers the safety risk of collision avoidance, but does not consider the impact of dynamic factors on stability. At the same time, stability has a certain impact on tracking control accuracy, which should also be considered. Therefore, the contribution of this paper is to propose a real-time emergency steering collision avoidance and stability control method, and design a simulation experiment based on the influence of stability control on tracking accuracy and other factors.

**FIGURE 1:**
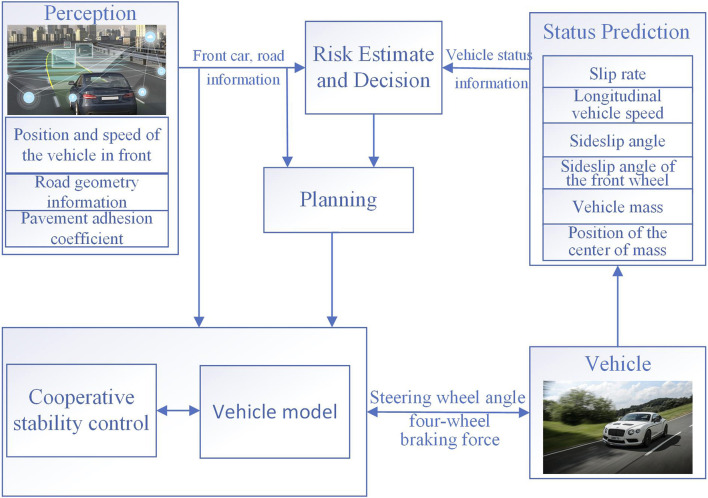
Coordinated control for vehicle driving stability and collision avoidance safety.

As shown in Figure 1, the lateral path planning and path tracking control considering motion stability designed in this paper are parts of the coordinated control architecture of vehicle driving stability and collision avoidance safety, which can realize automatic collision avoidance control and ensure the vehicle’s security and stability. Based on perception and state estimation information, the framework judges driving safety and collision risk and makes decisions based on TTC (time to collision), and uses dynamic programming and quadratic programming methods to plan paths and determine collision-free paths. Then, according to the characteristics of stability control in emergency collision avoidance scenarios, an adaptive MPC control algorithm is designed. Finally, the obtained steering wheel angle and four-wheel braking force are applied to the actual vehicle.

## Vehicle dynamics model

### 3-DOF vehicle dynamics model

**FIGURE 2:**
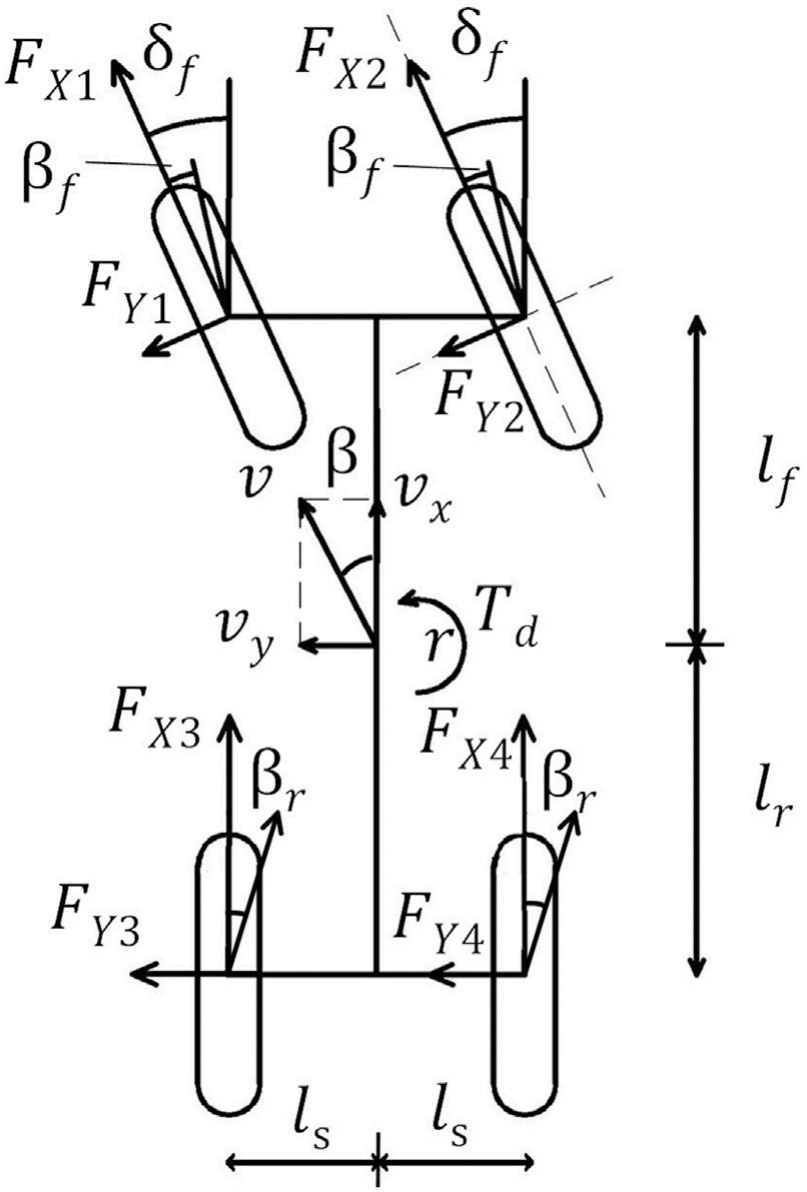
3-DOF vehicle dynamics model.

We rationally simplified the vehicle model (Wu G. 2021) to obtain a three-degree-of-freedom(3-DOF) vehicle dynamics model, as shown in Figure 2, which mainly includes vehicle longitudinal, lateral and yaw motions, where $\delta_{f}$ is the front wheel angle, and $F_{Xi}$ is the wheel braking force, $F_{Yi}$ is the wheel lateral force, $v_{x}$ is the longitudinal speed, $v_{y}$ is the lateral speed, v is the speed of the vehicle, β is the sideslip angle, $l_{f}$ and $l_{r}$ are the distance of the center of mass and the front and rear axles, $l_{s}$ is the wheelbase, $\beta_{f}$ is the sideslip angle of the front wheel, $T_{d}$ is the sum of the yaw moment of the vehicle, r is the yaw rate of the vehicle.

The differential equations of motion of the vehicle:

$$\left\{\begin{array}{c} \sum F_{xi}=m\left({\dot{v}}_{x}-r\times v_{y}\right)\\ \sum F_{yi}=m\left({\dot{v}}_{y}+r\times v_{x}\right)\\ l_{f}\left(F_{y1}+F_{y2}\right)-l_{r}\left(F_{y3}+F_{y4}\right)+l_{s}\left(F_{x1}+F_{x3}\right)-l_{s}\left(F_{x2}+F_{x4}\right)=T_{d} \end{array}\right.$$ (1)

So far, the establishment of the 3-DOF model of the vehicle considering the lateral, longitudinal and yaw motions has been completed, and this model will be used to describe the basic characteristics of the vehicle during motion.

### Linear tire model

In the case of a small vehicle front wheel angle, the relationship between the wheel lateral force and the sideslip angle of this wheel can be approximately regarded as a linear relationship (Wu G. 2021), thus:

$$\left\{\begin{array}{c} F_{Yi}=k_{f}\beta_{f}\;i=1,2\\ F_{Yi}=k_{r}\beta_{r}\;i=3,4 \end{array}\right.$$ (2)

Where $k_{f}$ and $k_{r}$ are the cornering stiffnesses of the front and rear axles, respectively. Front and rear wheel sideslip angles, vehicle sideslip angle and their derivatives are:

$$\left\{\begin{array}{c} \beta_{f}=\beta +\frac{l_{f}r}{v_{x}}-\delta_{f}\\ \beta_{r}=\beta -\frac{l_{r}r}{v_{x}} \end{array}\right.$$ (3)

$$\left\{\begin{array}{c} \beta =\frac{v_{y}}{v_{x}}\\ \dot{\beta }=\frac{\dot{v_{y}}v_{x}-\dot{v_{x}}v_{y}}{{v_{x}}^{2}} \end{array}\right.$$ (4)

## Collision avoidance trajectory planning

**FIGURE 3:**
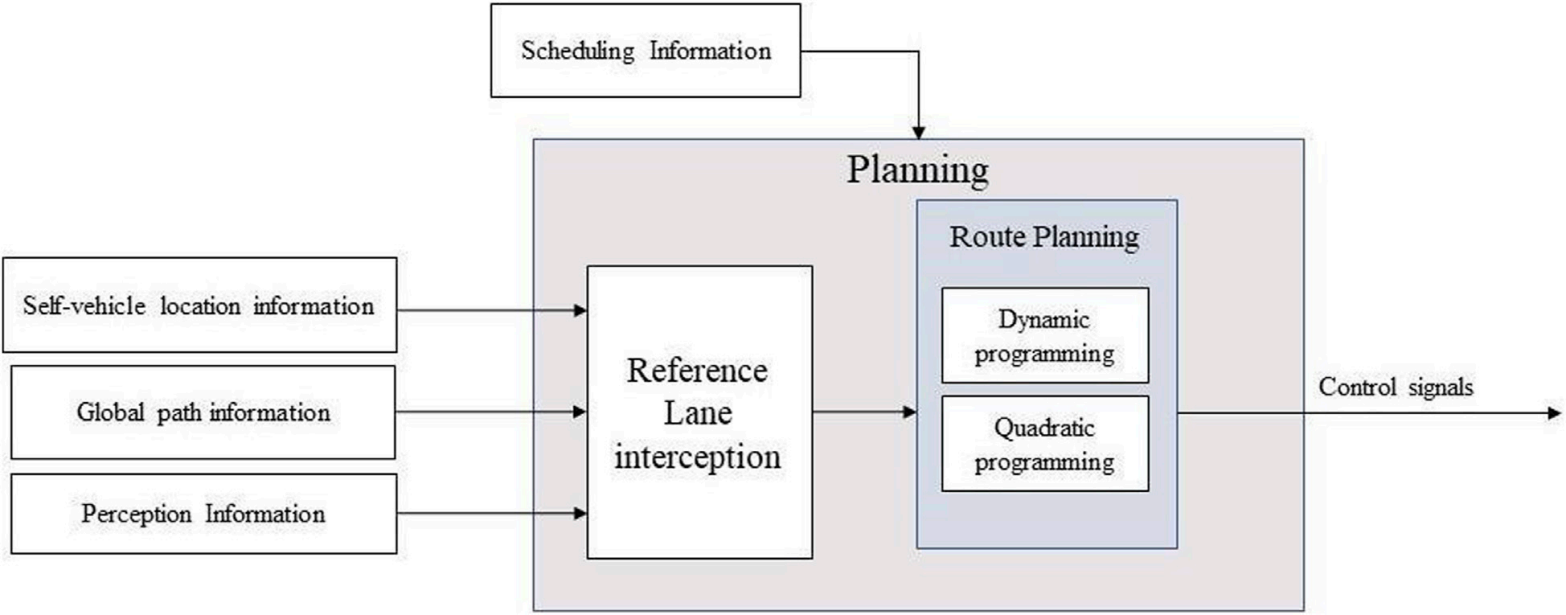
Trajectory planning during the steering.

The goal of trajectory planning is to generate a smooth enough curve to change the position of the vehicle under the premise of ensuring the safety of the vehicle. The smoothness is to ensure that the vehicle can track along the trajectory. The trajectory planning module will receive the environment information including vehicle location information and road information. A planned trajectory is transmitted to the vehicle motion control module as shown in Figure 3.

Usually trajectory planning can be decomposed into speed planning and path planning. Since the change of speed is not considered in the planning process of steering collision avoidance, we assume that the longitudinal speed is constant in the planning process, and only the path planning is designed here.

### Path planning

Using numerical methods, the path planning problem in discrete space can be abstracted as a quadratic programming problem, and the construction of this problem mainly includes two parts: the design cost function and the determination of the constraints of the problem. In designing the cost function, we need to consider the requirements of smoothness, not deviating from the road centerline and being away from obstacles. At the same time, in order to accelerate the solution, we first use dynamic programming to open up feasible space and therefore determine the constraints of the planning problem. After the problem is constructed, we use the iterative method to solve the quadratic programming problem.

The cost function of path planning can be divided into smoothing cost, reference line cost and obstacle cost. The road is discretized along the centerline and its perpendicular direction, x is the coordinate of the road centerline, and y is the coordinate of the point which is perpendicular to the road.

The smoothing cost $Cp_{smooth}$ is divided into three parts, $Wp_{smooth1}$ , $Wp_{smooth3}$ , and $Wp_{smooth3}$ , and their meanings correspond to the cost weights generated by the first, second, and third derivatives of the path:

$$Cp_{smooth}=Wp_{smooth1}y\prime^{T}y,+Wp_{smooth2}y{\prime \prime }^{T}y^{\prime \prime }+Wp_{smooth3}y^{\prime \prime }\prime^{T}y\prime \prime \prime$$ (5)

The reference line cost is $Cp_{ref}$ , and $Wp_{ref}$ represents the corresponding weight:

$$Cp_{ref}=Wp_{ref}y^{T}y$$ (6)

The obstacle cost is $Cp_{collision}$ , $Wp_{collision}$ represents the corresponding weight, and d represents the distance between the obstacle and the vehicle, where $Wp_{collision}$ is a rather large value.

$$Cp_{collision}=\left\{\begin{array}{c} 0\;,if\;d\geq 4\\ \frac{1000}{d}\;\;,if\;3<d<4\\ Wp_{collision},if\;d\leq 3 \end{array}\right.$$ (7)

Combining the above three formulas, the total planning cost $Cp_{node}$ of each discrete point can be obtained, and its value is equal to the sum of the above three costs:

$$Cp_{node}=Cp_{smooth}+Cp_{ref}+Cp_{collision}$$ (8)

The planned trajectory needs that the curvature is continuous. Δx is the sampling interval in the direction of the road centerline, ${y_{i}}^{\prime }$ is the first derivative of y to x at the i-th sampling point, ${y_{i}}^{\prime \prime }$ is the second derivative of y to x at the i-th sampling point:

$${y_{i+1}}^{\prime }={y_{i}}^{\prime }+{y_{i}}^{\prime \prime }\Delta x+\frac{1}{2}{y_{i}}^{\prime \prime }\Delta x^{2}+\frac{1}{2}\left(\frac{{y_{i+1}}^{\prime \prime }-{y_{i}}^{\prime \prime }}{\Delta x}\right)\Delta x^{2}$$ (9)

**FIGURE 4:**
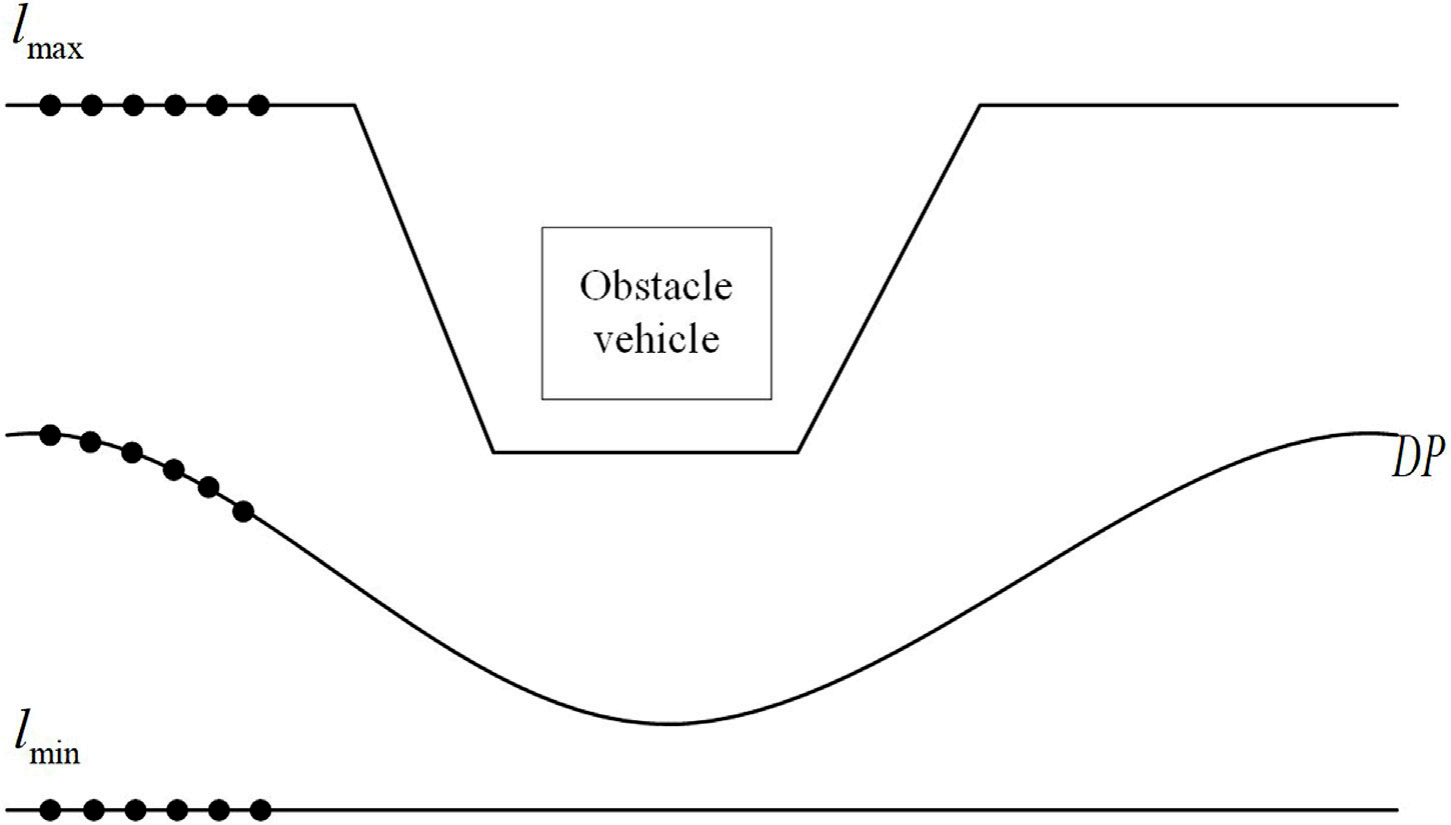
Path planning feasible space(DP).

The solution of the planning is first to obtain the initial solution by dynamic programming, and then to obtain the final result by secondary programming, as shown in Figure 4. In order to speed up the calculation of the quadratic programming problem, the result of the dynamic programming is regarded as a rough solution and a feasible space is opened up. $y_{\max\left(j\right)}$ , $y_{\min\left(j\right)}$ is the maximum and minimum value of the feasible space, $y_{\min\left(j\right)}^{\prime },y_{\max\left(j\right)}^{\prime }$ is the value range of the path restricted by the road, y_obs is the position corresponding to the obstacle car, width/2 is the width of the obstacle car:

$$\left\{\begin{array}{c} y_{\max\left(j\right)}=\min\left(y_{\max\left(j\right)}^{\prime },y\_obs-width/2\right),if\;y_{dp\_path}>y\_obs\\ y_{\min\left(j\right)}=\max\left(y_{\min\left(j\right)}^{\prime },y\_obs+width/2\right),others \end{array}\right.$$ (10)

After a lot of tuning, the parameters of the planning algorithm are finally selected $Wp_{smooth1}=15;Wp_{smooth2}=20000;Wp_{smooth3}=5000$ ; $Wp_{ref}=15$ .

### Quadratic programming problem solving

The advantage of Dynamic Programming (DP) is to decompose each column of the discrete space into a sub-problem and solve the optimal path from the last column through the inverse method. On the basis of discretization of the solution space, the initial path can be calculated by using dynamic programming. According to this path, a preliminary decision can be made on the path planning problem to reduce the search range of the quadratic feasible space, as shown in Figure 4. The advantage of the iterative method for solving quadratic programming problems is that it can balance the solution time and effect. This article does not focus on this aspect, so directly call the quadratic planner solution function in MATLAB.

## Control strategy of collision avoidance system

**FIGURE 5:**
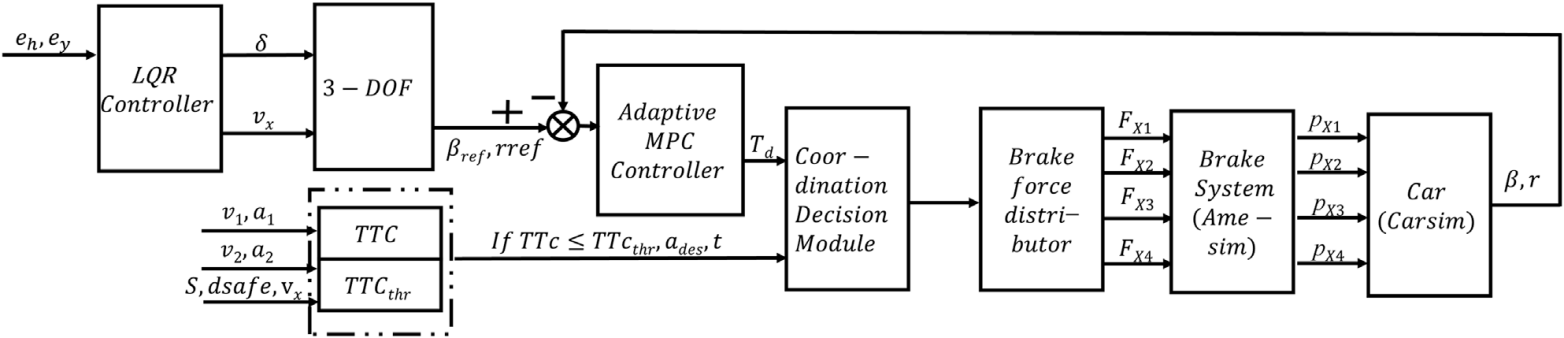
Vehicle emergency steering and collision avoidance stability control.

In this paper, a path-following control system considering motion stability is proposed. Its purpose is to judge the risk of collision when an obstacle appears in front of the vehicle, and automatically implement emergency collision avoidance with the stability of the vehicle body, as shown in Figure 5. The structure of the system can be mainly divided into three parts: TTC risk assessment, LQR lateral control and adaptive Model Predictive Control (MPC) stability control.

First, according to the collision risk assessment module, the collision time TTC is calculated according to the state of the vehicle and the environment perception information to judge the safety of the current vehicle. TTC refers to the time it would take for a collision to occur at the prevailing speeds, distances, and trajectories associated with the driver’s vehicle and the closest lead vehicle. TTC can be kinematically defined as the range between a following and lead vehicle divided by the relative velocity between these vehicles. Hence, TTC provides a measure of crash risk or the time before impact if prevailing conditions continue (Coelingh et al., 2010; Han et al., 2014). We have carried out related research in this part, but it is not the focus of this article. Considering the complex and changeable traffic conditions, as well as the possible instability of the vehicle caused by the large action of the actuator, this paper designs an adaptive MPC module to control the yaw moment of the vehicle to ensure the stability of the vehicle. The module judges whether to intervene. In addition, the linear quadratic regulator (LQR) controller is proposed in this paper to calculate the output signal steering angle δ according to the lateral error $e_{h}$ and heading angle error $e_{y}$ , so that the vehicle can always track the road centerline. Finally, in terms of braking force control and distribution, the braking force distribution and control module will calculate the braking forces $F_{X1}、F_{X2}、F_{X3}、F_{X4}$ according to the expected deceleration and the expected additional yaw moment of the vehicle, and From this, the braking pressures $P_{X1}、P_{X2}、P_{X3}、P_{X4}$ of each wheel cylinder are further obtained.

### LQR lateral control

The main purpose of lateral control is to control the lateral error within a certain range. As a result we can get a better track of the desired path and the heading angle of the vehicle. The content of this section is mainly based on the LQR to design the controller to track path. The state variables of the control system are four parameters, including: lateral error $e_{y}$ , rate of change of lateral error $\dot{e_{y}}$ , and heading angle error $e_{h}$ , the rate of change of heading angle error $\dot{e_{h}}$ . The following formula is the state space equation of the system:

$$\left[\begin{array}{c} \dot{e_{y}}\\ {ë}_{y}\\ \dot{e_{h}}\\ {ë}_{h} \end{array}\right]=\left[\begin{array}{cccc} 0 & 1 & 0 & 0\\ 0 & \left.{\frac{1}{mu}(k}_{f}+k_{r}\right) & -\frac{1}{m}\left(k_{f}+k_{r}\right) & \frac{1}{mu}\left(l_{f}k_{f}-l_{r}k_{r}\right)\\ 0 & 0 & 0 & 1\\ 0 & \frac{1}{I_{z}v_{x}}\left(l_{f}k_{f}-l_{r}k_{r}\right) & -\frac{1}{I_{z}}\left(l_{f}k_{f}-l_{r}k_{r}\right) & \frac{1}{I_{z}v_{x}}\left({l_{f}}^{2}k_{f}+{l_{r}}^{2}k_{r}\right) \end{array}\right]\left[\begin{array}{c} e_{y}\\ \dot{e_{y}}\\ e_{h}\\ \dot{e_{h}} \end{array}\right]+\left[\begin{array}{c} 0\\ -\frac{k_{f}}{m}\\ 0\\ -\frac{ak_{f}}{I_{z}} \end{array}\right]\delta_{f}+\left[\begin{array}{c} 0\\ \frac{1}{mv_{x}}\left(ak_{f}-bk_{r}\right)-v_{x}\\ 0\\ \frac{1}{I_{z}v_{x}}\left(a^{2}k_{f}+b^{2}k_{r}\right) \end{array}\right]\dot{\delta_{f}}$$ (11)

Where:

$$A=\left[\begin{array}{cccc} 0 & 1 & 0 & 0\\ 0 & \left.{\frac{1}{mv_{x}}(k}_{f}+k_{r}\right) & -\frac{1}{m}\left(k_{f}+k_{r}\right) & \frac{1}{mv_{x}}\left(l_{f}k_{f}-l_{r}k_{r}\right)\\ 0 & 0 & 0 & 1\\ 0 & \frac{1}{I_{z}v_{x}}\left(l_{f}k_{f}-l_{r}k_{r}\right) & -\frac{1}{I_{z}}\left(l_{f}k_{f}-l_{r}k_{r}\right) & \frac{1}{I_{z}v_{x}}\left({l_{f}}^{2}k_{f}+{l_{r}}^{2}k_{r}\right) \end{array}\right],B=\left[\begin{array}{c} 0\\ -\frac{k_{f}}{m}\\ 0\\ -\frac{ak_{f}}{I_{z}} \end{array}\right],C=\left[\begin{array}{c} 0\\ \frac{ak_{f}-bk_{r}}{mv_{x}}-\delta_{f}\\ 0\\ \frac{a^{2}k_{f}+b^{2}k_{r}}{I_{z}v_{x}} \end{array}\right]$$ (12)

Eq.11 can be expressed as:

$$\dot{e_{rr}}=Ae_{rr}+Bu+C\dot{\theta_{r}}$$ (13)

Error:

$$e_{rr}={\left[\begin{array}{cc} \begin{array}{ccc} e_{y} & \dot{e_{y}} & e_{h} \end{array} & \dot{e_{h}} \end{array}\right]}^{T}$$ (14)

The control quantity is $\delta_{f}$ .

From the above state equation, we can get the objective function and corresponding constraints of the LQR controller:

$$\mathrm{Min}\;J=\frac{1}{2}\int_{t_{0}}^{t_{f}}({e_{rr}\left(t\right)}^{T}Q\;e_{rr}(t)+{u\left(t\right)}^{T}R\;u(t)dts.t.\;\dot{e_{rr}}=Ae_{rr}\left(t\right)+Bu$$ (15)

Where $\left[t_{0},t_{f}\right]$ is the time domain, Q and R represent the weighted matrix of state and control quantity.

The optimal solution of this problem satisfies the following:

$$J^{*}={e_{rr}\left(t\right)}^{T}P\;e_{rr}\left(t\right)$$ (16)

The expression of P is:

$$\dot{P}=P\left(t\right)\;A\left(t\right)+A^{T}\left(t\right)\;P\left(t\right)-P\left(t\right)B\left(t\right)R^{-1}\left(t\right)B^{T}\left(t\right)P\left(t\right)+Q\left(t\right)$$ (17)

The LQR controller is:

$$u_{k}=-K\left(t\right)\;X\left(t\right)$$ (18)

Where $K=-R^{-1}\left(t\right)B^{T}\left(t\right)\;P\left(t\right)$ represents the controller gain.

### Adaptive model predictive control stability control module

In order to trade off the calculation efficiency and calculation accuracy, we assume that the longitudinal velocity $v_{x}$ in the Formula. 1 of the vehicle dynamics model remains unchanged. At this time, the three-degree-of-freedom model of the vehicle is simplified to two-degree-of-freedom. At the same time, it is brought into the Formula. 4, where the longitudinal velocity $v_{x}$ is a time-varying model parameter, and the sideslip angle and the yaw rate are taken as the state quantities, which can be finally simplified to obtain the following state space equation:

$$\left[\begin{array}{c} \dot{r}\\ \dot{\beta } \end{array}\right]=\left[\begin{array}{c} \begin{array}{cc} \frac{{l_{f}}^{2}k_{f}+{l_{r}}^{2}k_{r}}{v_{x}I_{z}} & \frac{l_{f}k_{f}-l_{r}k_{r}}{I_{z}} \end{array}\\ \begin{array}{cc} \frac{k_{f}l_{f}-k_{r}l_{r}}{m{v_{x}}^{2}}-1 & \frac{k_{f}+k_{r}}{mv_{x}}-\frac{\dot{v_{x}}}{v_{x}} \end{array} \end{array}\right]\left[\begin{array}{c} r\\ \beta \end{array}\right]+\left[\begin{array}{c} \frac{1}{I_{z}}\\ 0 \end{array}\right]T_{d}+\left[\begin{array}{c} -\frac{l_{f}k_{f}}{I_{z}}\\ -\frac{k_{f}}{mu} \end{array}\right]\delta_{f}$$ (19)

Where select $x={\left[\begin{array}{cc} r & \beta \end{array}\right]}^{T}$ as the state quantity, and $u=T_{d}$ as the control quantity.

Eq. 9 will calculate the reference values of the vehicle’s yaw rate and sideslip angle. The deviation value is used as the index of vehicle lateral stability, and the larger the value, the greater the risk of lateral instability of the vehicle.

$$\left\{\begin{array}{c} r_{ref}=\frac{v_{x}/\left(l_{f}+l_{r}\right)}{1+K{v_{x}}^{2}}\delta_{f}\\ \beta_{ref}=\frac{l_{r}+ml_{f}{v_{x}}^{2}/\left(k_{r}l_{f}+k_{f}l_{r}\right)}{\left(l_{f}+l_{r}\right)\left(1+K{v_{x}}^{2}\right)}\\ K=\frac{m}{{\left(l_{f}+l_{r}\right)}^{2}}\left(\frac{l_{f}}{k_{r}}-\frac{l_{r}}{k_{f}}\right) \end{array}\right.$$ (20)

$r_{ref}$ , $\beta_{ref}$ are the reference yaw rate and sideslip angle, respectively, and K is the stability factor.

MPC is a feedback control strategy that discretizes the vehicle dynamics equation, and sets the sampling time as $T_{s}=5\times 10^{-4}s$ . For time k, there is the following discrete equation:

$$\left\{\begin{array}{c} x\left(k+1\right)=Ax\left(k\right)+B_{u}u\left(k\right)+B_{d}d\left(k\right)\\ y\left(k\right)=Cx\left(k\right)+Du\left(k\right) \end{array}\right.$$ (21)

Where,

$$A=T_{s}*A_{c}+I,B_{u}=B_{cu}T_{s},B_{d}=B_{cd}T_{s},C=C_{c},D=D_{c}$$

$x\left(k\right),u\left(k\right),y\left(k\right)$ represent the state, control input and output of the system at time k, respectively. Assuming that the prediction time domain is $p_{s}$ , and the control time domain is $m_{s}$ , the control quantity will not change when the time exceeds the control time domain. Then the input and output predicted by time k are:

$$\left\{y_{p}\left(k+1|k\right),y_{p}\left(k+2|k\right),...,y_{p}\left(k+p_{s}|k\right)\right\}$$ (22)

$$\left\{u\left(k|k\right),u\left(k+1|k\right),...,u\left(k+p_{s}-1|k\right)\right\}$$ (23)

The control goal is to track the target and reduce the tracking error, that is,

$$\left\{r\left(k+1\right),r\left(k+2\right),...,r\left(k+p_{s}\right)\right\}$$ (24)

At the same time, the control constraints and output constraints of the system are set. Finally the optimization goal function that can characterize the control performance of the system is proposed. It needs to consider the cost of the expected tracking error and some other performances, such as the control action as small as possible. The objective function is:

$$J\left(y\left(k\right),U\left(k\right)\right)=\overset{p_{s}}{\underset{i=1}{\sum }}{\left\|\Gamma_{y}\left(r\left(k+i\right)-y_{p}\left(k+i|k\right)\right)\right\|}_{2}+{\overset{m_{s}}{\underset{i=1}{\sum }}\left\|\Gamma_{u}\left(u\left(k+i|k\right)-u\left(k+i-1|k\right)\right)\right\|}_{2}$$ (25)

$\Gamma_{y}$ is the weight of the output quantity, and $\Gamma_{y}$ u is the weight of the control quantity increment. For time k, the open-loop optimization problem is transformed into solving $\min\left(J\left(x\left(k\right),U\left(k\right),m_{s},p_{s}\right)\right)$ for the control variable U.

In order to improve the calculation efficiency, the longitudinal velocity $v_{x}$ is assumed to be constant when calculating the state space equation, but in the process of emergency collision avoidance, its longitudinal velocity $v_{x}$ is a time variable. At this time, the internal model of MPC will also change with time, so an adaptive MPC solution method is proposed. The longitudinal velocity and longitudinal acceleration output by the system are fed back to the MPC controller to update the internal model of the controller, which is beneficial to improve controller performance.

After a large number of parameter tuning and system identification, the system can maintain the best performance as much as possible, and the corresponding parameters of the adaptive MPC controller are selected: prediction time domain $p_{s}$ = 10, control time domain $m_{s}$ = 5, and add hard constraints to the control input, Take $u_{\min}=4000Nm$ , $u_{\max}=4000Nm$ , $\Delta u_{\max}=\pm 1000Nm$ , $\Gamma_{u}=0.02$ , $\Gamma_{y}=\left[\begin{array}{cc} 1 & 2 \end{array}\right]$ .

So far, the parameter setting of the adaptive MPC controller is completed, and the optimal additional yaw moment ΔM can be calculated to avoid vehicle instability.

### Vehicle tire braking force distribution strategy

**FIGURE 6:**
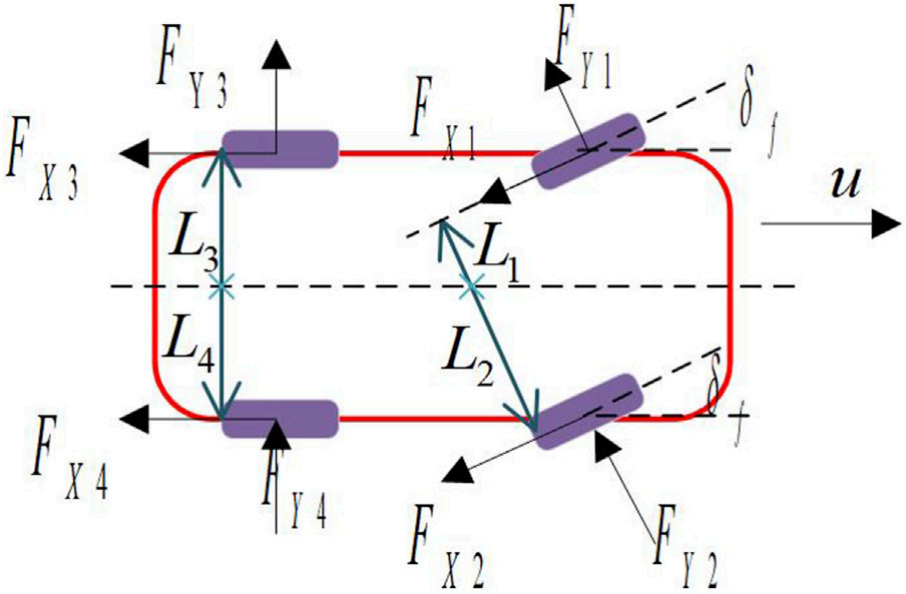
Four-wheel braking force distribution.

The additional yaw moment $T_{d}$ required by the vehicle has been obtained from the MPC controller as shown in Figure 6:

$$T_{d}=\overset{4}{\underset{i=1}{\sum }}{\left(-1\right)}^{i}F_{xi}L_{i}$$ (26)

$F_{ri},F_{fi}$ are the longitudinal forces of the front and rear tires, $d_{ri},d_{fi}$ are the lateral distances from the front and rear tires to the center of gravity of the vehicle. I represents the left or right side of the vehicle.

At present, the braking force distribution schemes for active braking of vehicles can be roughly divided into two types, single-wheel braking and multi-wheel braking. The braking force provided by the multi-wheel braking scheme is greater than that of the single-wheel braking scheme, but at the same time, the impact generated by the double-wheel braking scheme in the active braking process is also relatively large. In addition, the additional yaw moment generated by the wheel braking scheme is also larger. Therefore, in the face of emergencies, a multi-wheel braking scheme with faster control speed and larger upper limit of additional yaw moment is often adopted.

During the turning, the effects of braking different wheels on the steering dynamic performance are different, and the single-wheel control strategy will select different wheels for control under different vehicle states. If it is a two-wheel braking scheme, when the car is about to flick or understeer, the system will adopt the method of active braking the two wheels on the outer side of the vehicle’s rotation direction at the same time to adjust the body state. The two wheels on the inner side of the steering wheel perform active braking to correct the body condition.

Then, it is necessary to select the most effective wheel to generate $T_{d}$ according to the actual situation. If the front wheels cannot provide enough additional yaw moment, the remaining yaw moment can be generated by the training wheels. In order to design the wheel selection strategy, define the following formula:

$$ƛ={ƛ}_{M}\cdot {ƛ}_{\gamma },\left({ƛ}_{M}=sgn\left(T_{d}\right),{ƛ}_{\gamma }=sgn\left(r\right)\right)$$ (27)

**FIGURE 7:**
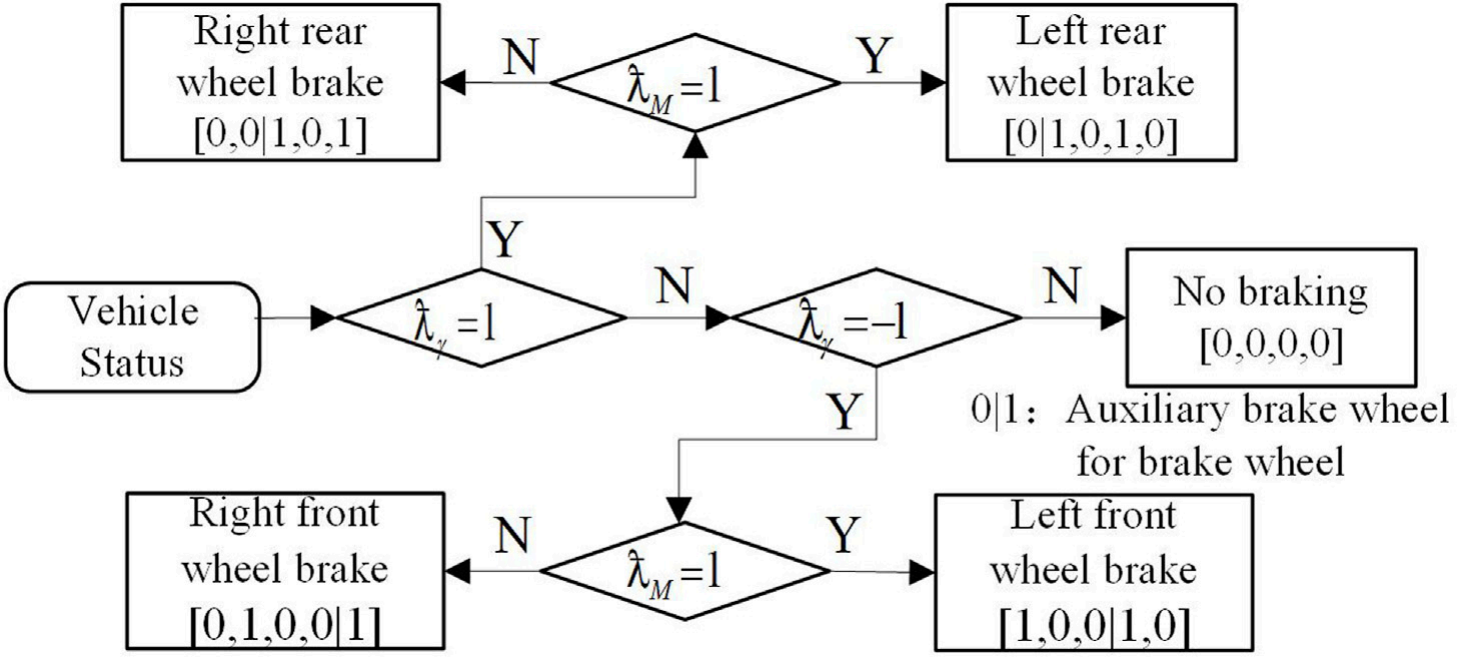
Tire selection strategy.

As shown in Figure 7, the wheel selection strategy is proposed based on ${ƛ}_{M}$ and ${ƛ}_{\gamma }$ . In Figure 7 [left front, right front wheel, rear left and right rear wheel], when the value in the vector is set to 1, corresponding tire brakes, when the value in the vector is set to 0, tire don’t brake, 0|1 said whether need the auxiliary brake wheel brake, for example, if the right front wheel braking does not produce enough yawing moment, we need to brake the right rear wheel to generate enough yaw torque. If the vehicle is oversteering (*ƛ*<0), the priority braking wheels are the front outer wheels of the vehicle. When the vehicle understeers (*ƛ*>0), the priority braking wheel is the rear inner wheel. Once the yaw moment provided by the front wheels is not enough, the remaining yaw moment can be generated by the selected auxiliary wheels.

Finally, according to the selected braking wheels, the distributed four-wheel braking force can be obtained from Formula. 26.

## Simulation experiments

**FIGURE 8:**
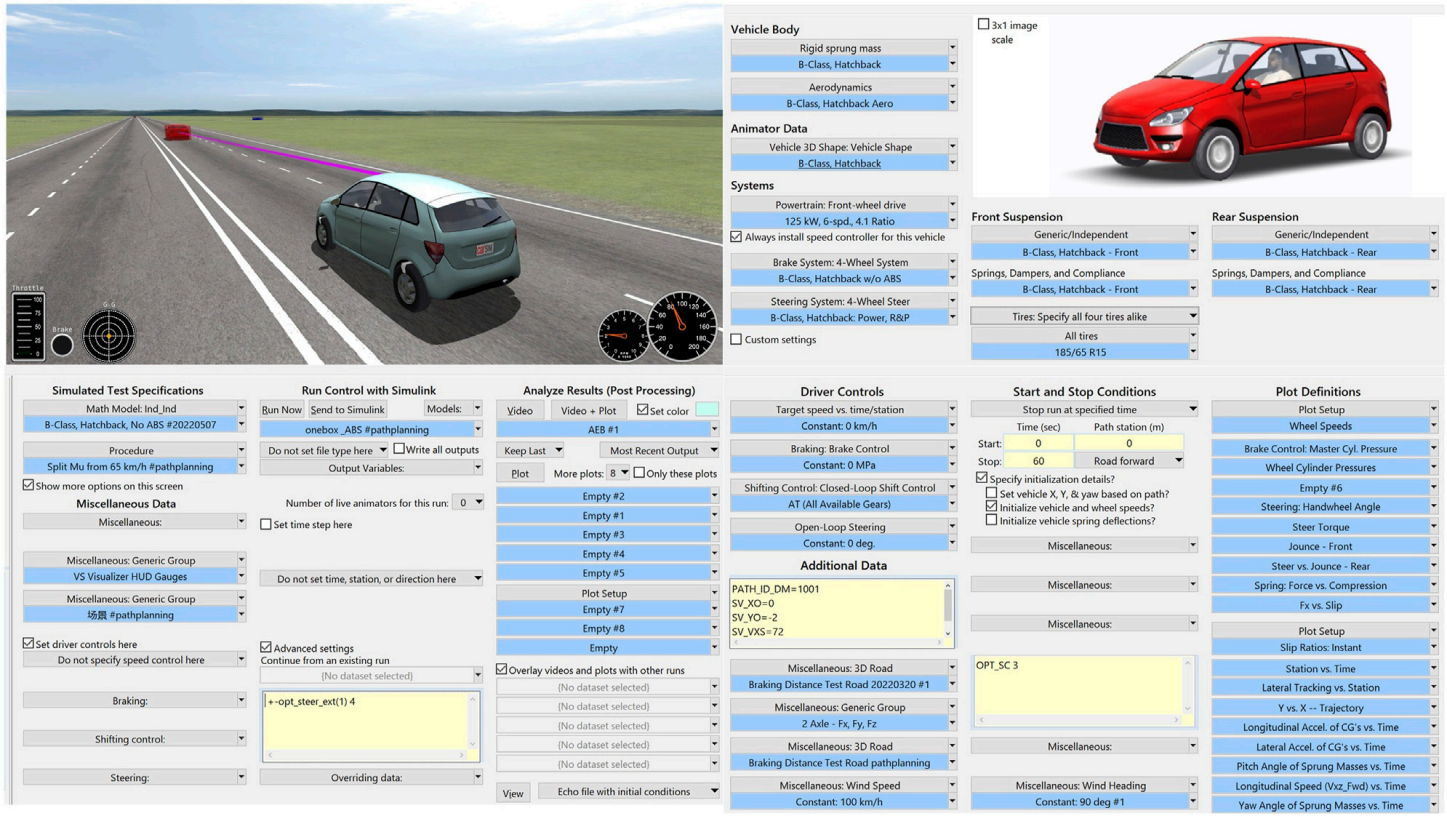
Carsim simulation diagram.

**FIGURE 9:**
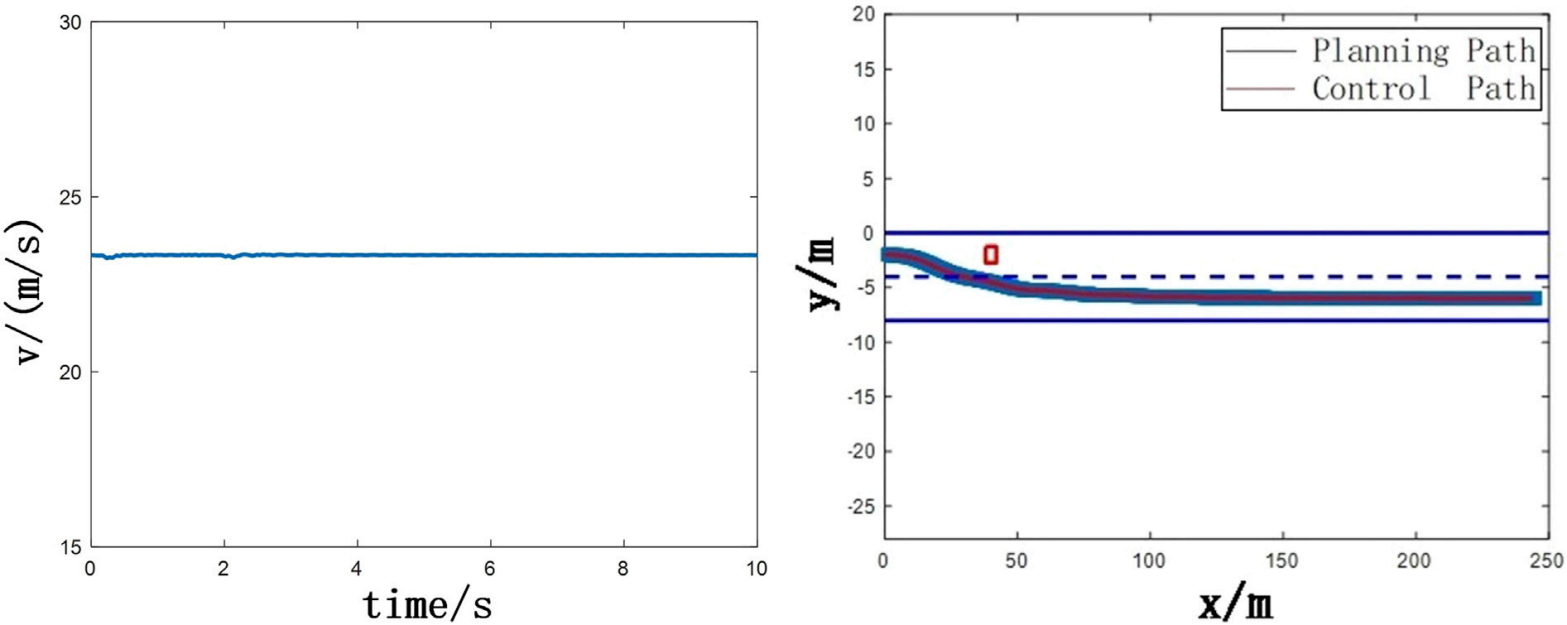
**(A)** Ego vehicle speed **(B)** Obstacle avoidance trajectory.

In order to verify the effectiveness of the algorithm in this paper, a joint simulation model was built in Carsim and MATLAB/Simulink, as shown in Figure 8. During the operation of the collision avoidance algorithm, the vehicle is driving at a speed of about 84 km/h. The obstacle car is located at (40, −2) position, as shown in Figure 9B. At this time, the vehicle is about to have a frontal collision, and the collision avoidance algorithm starts to run and the steering wheel is turned to the right. As shown in Table 1, the simulated vehicle parameters are selected from Carsim.

**TABLE 1:** Vehicle parameters.

Parameters	Units	Value
mass	kg	1570
The moment of inertia of the body around the *z*-axis	$kg∙m^{2}$	4192
Vehicle center of mass to front axle distance	m	1.04
Vehicle center of mass to rear axle distance	m	1.56
Vehicle front axle cornering stiffness	Nm/rad	−78329
Vehicle rear axle cornering stiffness	Nm/rad	−78329

### Without the stability control

**FIGURE 10:**
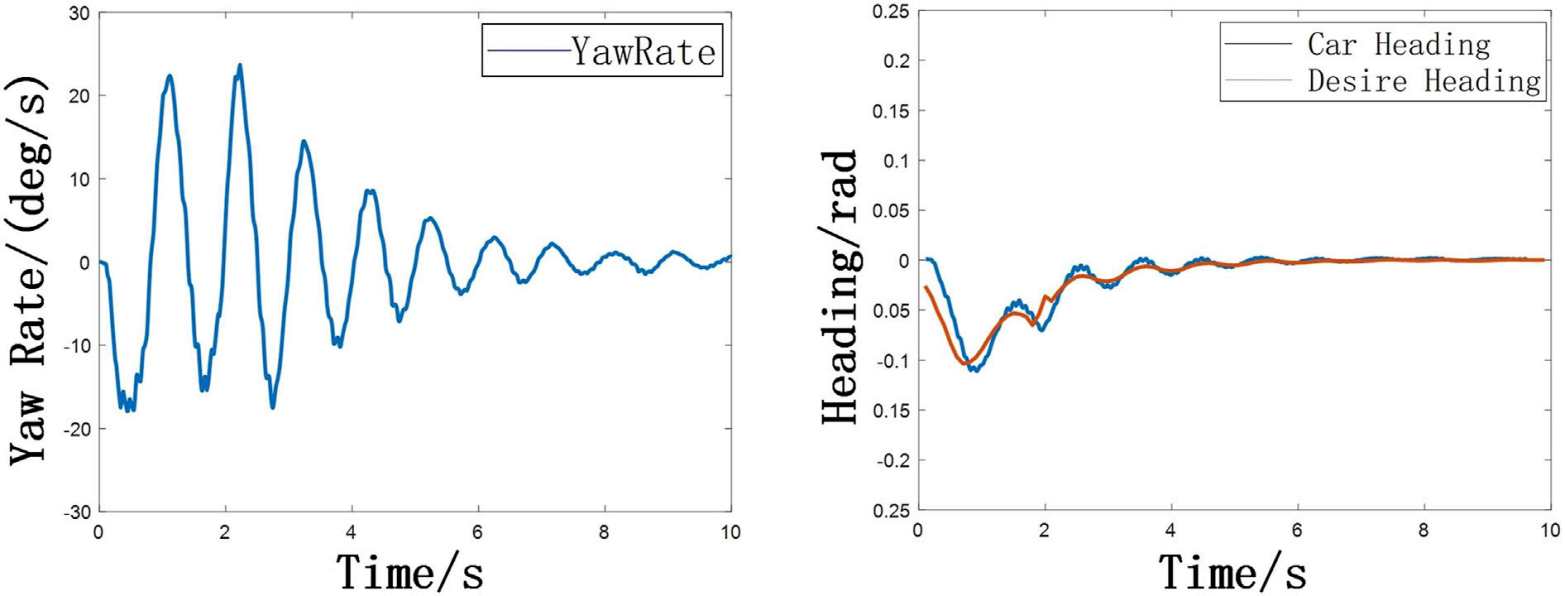
**(A)** Variation of yaw rate with time **(B)** Variation of heading angle with time.

Without the stability control, the planning algorithm and the lateral control algorithm as shown in Figures 9, 10 perceive that there is a slow-moving car at a speed of 10 km/h 40 m ahead, and then start to perform lane change to avoid obstacles. After about 2 s, the ego vehicle avoids obstacles, and in about 6 s, the heading angle gradually stabilizes near 0, but the yaw angle speed stabilizes slowly. At this time, the longitudinal velocity $v_{x}$ of the ego vehicle remains basically unchanged.

**FIGURE 11:**
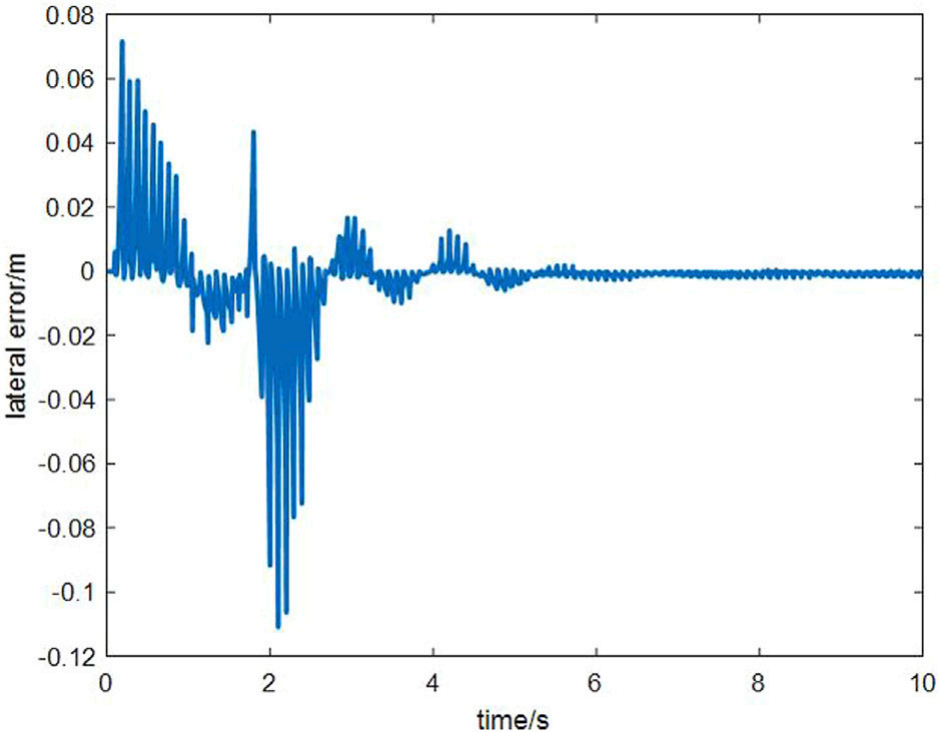
Variation of lateral error with time.

The tracking performance can be represented by the change of lateral error (The distance between the vehicle and its projection to the planned path) with time, as shown in the following Figure 11.

### With the stability control

**FIGURE 12:**
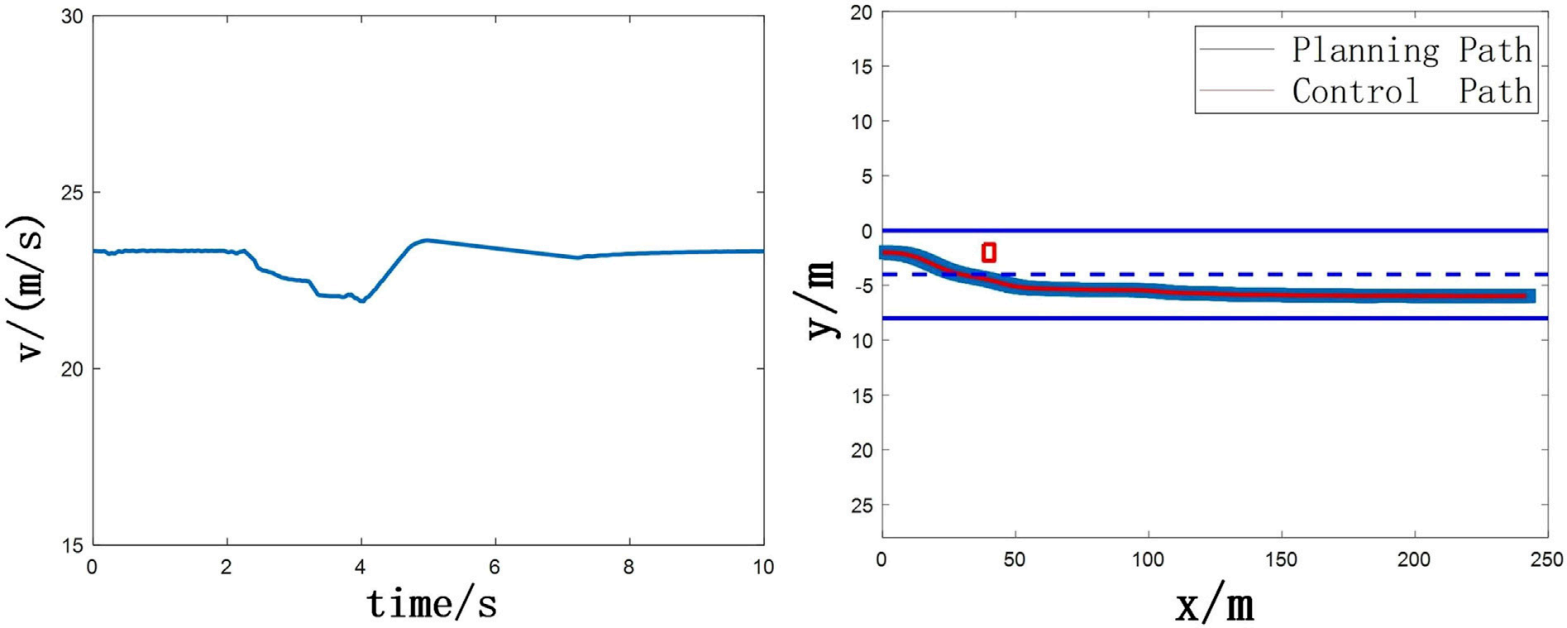
**(A)** Vehicle speed **(B)** Obstacle avoidance trajectory.

**FIGURE 13:**
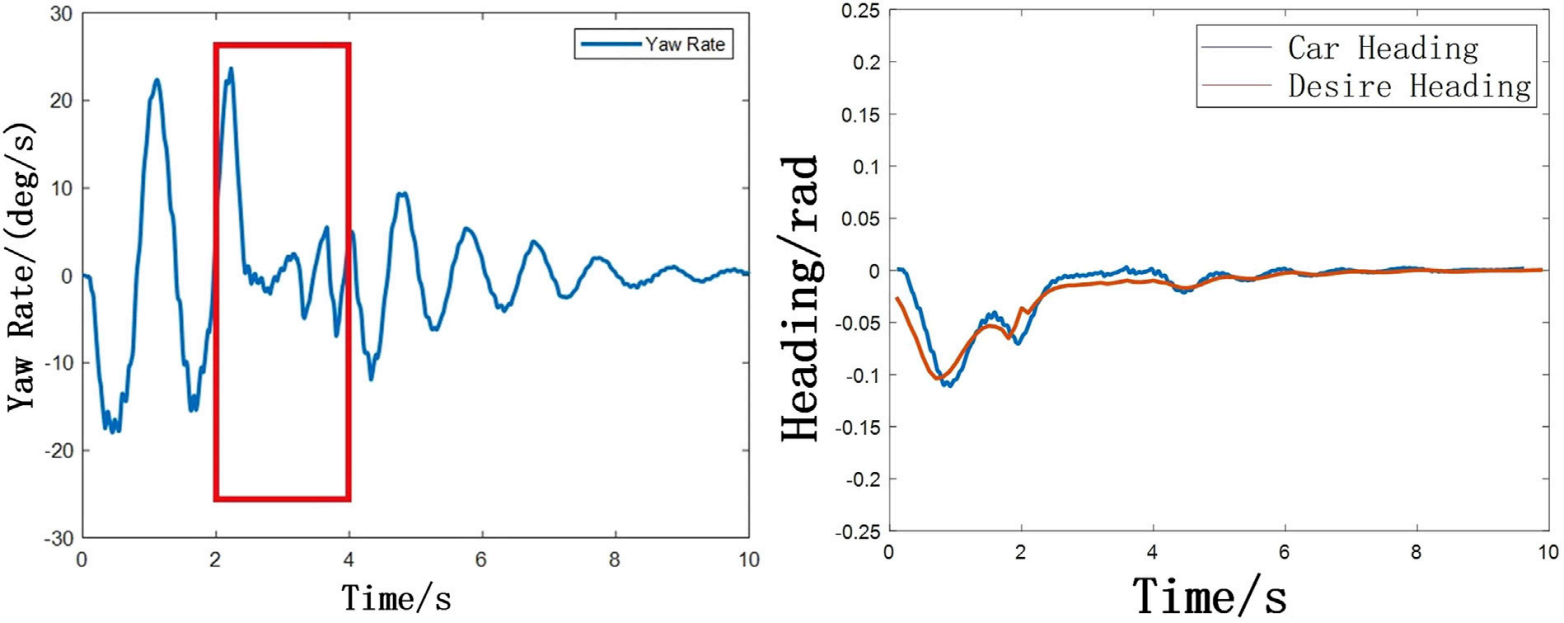
**(A)** Changes of yaw rate with time **(B)** Changes of heading angle with time.

**FIGURE 14:**
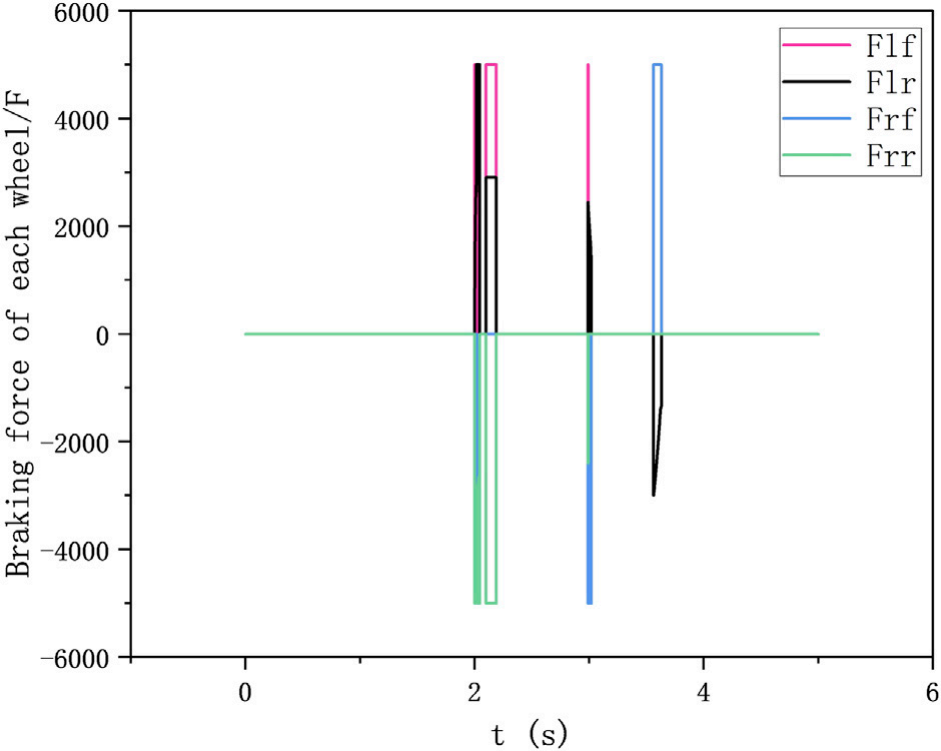
Change of tyre braking force with time.

The stability control intervenes within 2–4 s after the vehicle avoids the obstacle as shown in Figures 12, 13. At this time, the change of the yaw rate is quickly suppressed. At the same time, it can be seen that the heading angle stabilizes rapidly to 1° in 2.5 s. But there is a certain heading angle error. The reason why 2–4 s is chosen is due to the consideration of stability control on path tracking accuracy. Firstly, before avoiding obstacles (that is, before 2 s, this time is calculated by TTC), the stability control has a certain impact on obstacle avoidance, and may even lead to accidents, which should be avoided as much as possible. Secondly, if the stability control intervention time is too long or the stability control intervenes when the yaw angle is large, the lateral control algorithm cannot control the heading angle error to zero. This will cause the planning control algorithm to be unable to track the path stably according to the road centerline after the lane change. The change of tyre braking force with time as shown in Figure 14.

### Trajectory planning

**FIGURE 15:**
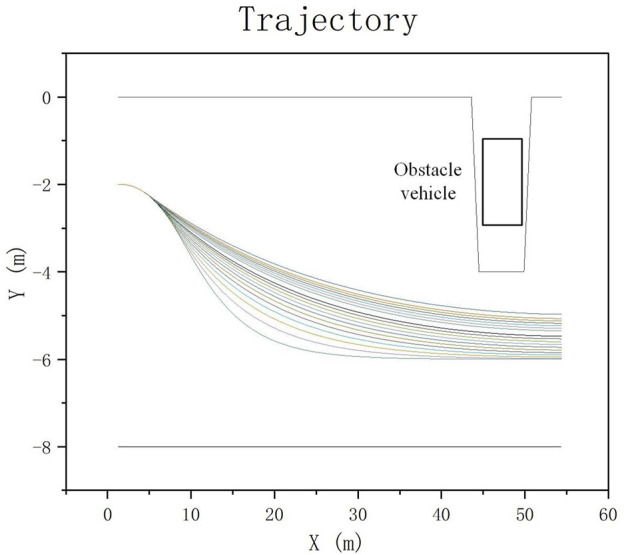
Trajectory planning.

On the premise of keeping other planning parameters consistent, we changed $Wp_{smooth2}$ from 1000 to 20,000 for a total of 20 tests (indicated by different colored trajectories). It can be seen that our algorithm successfully avoided the obstacle car ahead under the condition of considering the smooth trajectory as shown in Figure 15, which proves the effectiveness of our algorithm.

## 6 Conclusion

In this paper, the planning algorithm during steering and collision avoidance is studied based on the information and data given by vehicle environmental information perception and vehicle state parameter estimation The coordinated control of vehicle stability and safety is studied. The paper has carried out the following work: 1. Considering the calculation efficiency and control requirements of the model, a three-degree-of-freedom vehicle dynamics model and a linear tire model are established. 2. The planning module is proposed by the method of quadratic programming. This module will plan the driving trajectory of the vehicle by comprehensively considering the constraints and safety of vehicle execution. 3. Considering the vehicle trajectory tracking performance and stability, LQR lateral control and adaptive MPC control algorithms are proposed and the intervention time is proposed. 4. According to the results output by the MPC algorithm, the four-wheel braking force is distributed to realize the vehicle collision avoidance control under the comprehensive consideration of safety and stability. The results show that the planning algorithm in this paper can give a safe and reliable collision-free motion trajectory, and the proposed stability and safety coordination control algorithm can track the collision avoidance trajectory with high precision and stabilize the vehicle’s heading angle about 0.5s after avoiding obstacles.

## Data Availability

The original contributions presented in the study are included in the article/Supplementary Material, further inquiries can be directed to the corresponding author.
